# Vissage sacro-iliaque percutané

**DOI:** 10.11604/pamj.2015.22.372.8428

**Published:** 2015-12-16

**Authors:** Mohamed Amine Karabila, Ahmed Bardouni

**Affiliations:** 1 Service de Chirurgie Orthopédique et de Traumatologie, CHU Ibn Sina, Rabat, Maroc

**Keywords:** Vissage, luxation, percutanée, screw, dislocation, percutaneous

## Image en medicine

Nous rapportons le cas d'un patient âgé de 21 ans, victime d'une chute en moto. Le bilan radiologique objective une disjonction symphyse pubienne, une disjonction sacro iliaque gauche avec uneascension ouverture de l'hémi-bassingauche (A et B). Le patient a bénéficié au début de l'intervention d'une réduction de l'ascension de la sacro-iliaque par traction sur le membre puis ostéosynthèse première de la symphyse pubienne par une plaque de cotyle puis un vissage sacro-iliaque (C). Le vissage sacro-iliaque percutané nous semble une technique fiable et reproductible. Le traitement des fractures instables du bassin est pour nous la meilleure indication. La réduction des lésions avant toute tentative de vissage est indispensable. Si la réduction n'est pas obtenue, il est préférable de s'orienter vers une autre technique d'ostéosynthèse. La chirurgie doit se faire le plus rapidement possible après le traumatisme et si possible dans les 24 premières heures. A 12 mois de recul, le patient peut faire 10 km de marche avec 20 kilos dans le dos.

**Figure 1:**
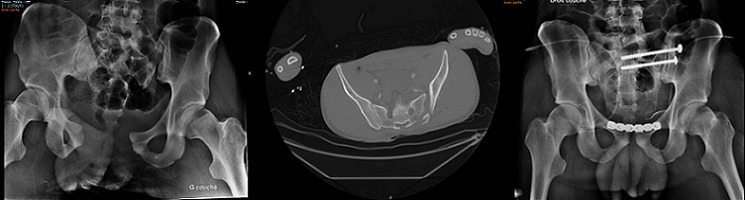
(A) radiographie du bassin montrant la disjonction de la symphyse pubienne et l'ascenssion de l'hémibassin gauche; (B) scanner montrant la disjonction sacro-iliaque gauche; (C) radiographie de bassin de contrôle

